# Are former heavy drinkers in the UK less likely to identify as being in recovery compared to those in the USA? A pilot test

**DOI:** 10.1186/s13011-021-00412-8

**Published:** 2021-09-26

**Authors:** John A Cunningham, Alexandra Godinho

**Affiliations:** 1 National Addiction Centre, Institute of Psychiatry, Psychology and Neuroscience, Kings College London, London, UK; 2 Centre for Addiction and Mental Health, Toronto, Canada; 3 Department of Psychiatry, University of Toronto, Toronto, Canada

**Keywords:** Alcohol, Natural history, Recovery identity, 12-step

## Abstract

**Background:**

To provide a preliminary test of the prediction that fewer former heavy drinkers will identify themselves as being in recovery in the UK versus the USA.

**Methods:**

An online cross-sectional survey was completed by a convenience sample of former heavy drinkers. This sample was identified from participants recruited to complete a questionnaire about alcohol consumption. The recruitment advertisement specified that the participants did not need to drink alcohol. The survey included items assessing self-reported current and past levels of alcohol consumption, alcohol dependence at time of heaviest alcohol consumption (ICD-10 criteria), and questions regarding identifying as currently or ever being in recovery taken from a survey by Kelly et al. (2018).

**Results:**

Out of 5002 participants who completed the questionnaire, 150 were identified as former heavy drinkers from the UK or the USA. The proportion of participants reporting alcohol dependence, and the proportion of participants reporting past year abstinence, did not differ significantly between the UK and the USA (*p* = .841 and 0.300 respectively). Compared to participants from the UK, participants in the USA were more likely to report that they had a problem with drinking but now no longer do (24.1 % vs. 56.0 %; *p* < .001), and that they currently identified (4.2 % vs. 21.2 %; *p* = .003) or ever identified (7.4 % vs. 30.2 %; *p* = .001) as being in recovery.

**Conclusions:**

Identifying as being in recovery appears more common in the USA than the UK among former heavy drinkers. This apparent difference in prevalence may reflect historic differences in treatment services offered in these countries, particularly with respect to the predominance of a 12-step approach in the USA. These findings should be replicated in a representative sample.

## Background

The term, ‘in recovery’ is used by the Alcoholics Anonymous (AA) movement to describe the ongoing need for vigilance to maintain sobriety (which includes being abstinent from alcohol consumption) and to prevent a relapse to hazardous drinking [1]. The person who adopts this term, or identity, may regard themselves as being in recovery for the rest of their life. While not explicitly defined in the AA literature, recovery is thought to involve improvements in health and wellbeing and has been described as a social process in which individuals learn to internalize new norms and values as part of a recovery-oriented identity [2–5]. The ‘in recovery’ term is also employed with the same meaning in the context of remissions from drug use or with other addictive behaviours, such as gambling [1].

While AA and the 12-step movement have a global reach, they largely originate in the USA (although its spiritual roots, as with the ancestors of the large majority of the US population, ‘migrated’ from other countries) [6]. As the recovery identity is particularly associated with AA and other 12-step movements, might the extent to which it is adopted by former heavy drinkers also vary by country? For example, the United Kingdom experienced a policy shift in the past decade leading to cuts in alcohol and drug treatment services, and to the orientation of services towards sobriety and away from harm reduction approaches [7–10]. With this adoption of a sobriety orientation being fairly recent, it may mean that that the recovery identify is not as common among former heavy drinkers in the UK compared to the US. This question is relevant because current and former heavy drinkers who are unwilling to adopt a recovery identity may be less likely to access, and benefit from, treatment programs employing a 12-step approach.

The current pilot study presents preliminary analyses to test the prediction that the recovery identity is more common amongst former heavy drinkers in the USA as compared to the United Kingdom. The study is regarded as a pilot primarily because the data is not representative of the general population. Nevertheless, the sample is adequate to provide a preliminary test of this prediction and to establish whether more research on this topic might be merited.

## Methods

The survey was conducted through the Prolific website and employed an advertisement asking for participants to take a short survey (approximately 15 min) about drinking alcohol [11]. The advertisement specified that participants did not need to be current or past drinkers. Participants were 18 years or older and there was no restriction regarding country of residence. Only those participants who were from the UK or the USA were retained for the current analyses. This decision was made, partly because of the purpose of this study (i.e., to compare participants from the UK and the USA) but also because there were insufficient numbers of participants recruited from other countries to include them as comparator groups. Country of residence was assessed directly rather than relying on pre-existing Prolific categories (i.e., after providing informed consent, the first question asked participants their country of residence).

The survey also included questions regarding current and past alcohol consumption that allowed us to identify participants who were former heavy drinkers using a set of criteria employed in previous research. Briefly, former heavy drinking was defined as endorsing having ever drunk five or more (USA; or six or more for the UK) drinks at least once a week for a month or longer, when participants were drinking at their heaviest. Of these participants, only those who reported no alcohol consumption in the past year (abstinent), or reported alcohol consumption of a lower risk (i.e., moderate drinking), were included in the former heavy drinking sample and only this subgroup was examined in subsequent analyses. The definition of current moderate drinking was: (1) usual consumption of two or less drinks per drinking day; (2) consuming five (six) or more drinks on one occasion less than once per month; and (3) having never consumed more than seven drinks (eight in the UK) on one occasion in the past 6 months [12–14].

Participants identified as former heavy drinkers were then asked several items taken from the Kelly et al. [15] survey study examining the proportion of former heavy drinkers (or drug users) in the USA who identified themselves as being in recovery. Specifically, the current study asked former heavy drinkers: (1) “Did you used to have a problem with alcohol but no longer do?”; (2) “Do you consider yourself to be in recovery?”; and (3) “Did you ever consider yourself to be in recovery?” Participants were then asked to endorse which of the following best described how much of a problem their drinking was at its heaviest – not at all, very minor, minor, major, very major or don’t know. Further, the severity of participants’ drinking was assessed using an eleven-item scale that measured ICD-10 alcohol dependence, framed to ask about the period when their drinking was at its heaviest [16].

At the end of the survey, and after answering questions asking about demographic characteristics, participants were asked whether they answered all the questions truthfully (1 = strongly disagree; 7 = strongly agree). In addition, an attention check question was nested in a series of questions early in the survey, “I want to indicate that I have read this question by checking all the time?” (‘all the time’ was one of the response options in this section of the survey) [17]. Only those who strongly agreed that they had answered the questions truthfully, and answered the attention check correctly, were retained in the sample.

## Results

A total of 5,002 participants completed the online survey, of which 4,450 strongly agreed that they had answered the questions truthfully and also answered the attention check question correctly. Of these 4,450 participants, 1250 stated that they resided in the UK and 746 in the USA. Of these 1,996 participants, 150 were defined as former heavy drinkers (97 from the UK and 53 from the USA). There were no significant differences (*p* > .05) in demographic characteristics between participants from the UK or the USA (see Table 1).

**Table 1:** Demographic characteristics of former heavy drinkers from the UK or the USA

	UK **(***n*** = 97)**	USA **(***n*** = 53)**	*p*
Mean (SD) Age	38.9 (11.4)	37.5 (11.2)	0.43
% Male	37.1	45.3	0.38
% Married/Common Law	57.7	49.1	0.39
% Completed education after turning 19	67.0	77.4	0.20
% Full/part time employed	69.1	79.2	0.25

Table 2 presents comparisons between former heavy drinking participants from the USA and the UK on their current drinking status (currently abstinent or moderate drinker), severity of their heaviest drinking period, rating of whether this past heavy drinking was a problem, and identification with being in recovery (currently or ever). The two countries did not differ significantly in the proportion of participants who were current moderate drinkers versus those who did not currently drink alcohol (*p* = .300). Further, there was no significant difference in the proportion of participants who met criteria for lifetime alcohol dependence (*p* = .841). However, compared to those in the UK, participants residing in the USA were more likely to state that they used to have a drinking problem on both the yes/no former problem question (24.1 % vs. 56.0 %; Fisher’s exact test, *p* < .001) and on the rating of severity of their drinking at its heaviest as a major or very major problem (10.3 % vs. 32.1 %; Fisher’s exact test, *p* = .002). Finally, compared to UK participants, those in the USA were more likely to identify as currently (4.2 % vs. 21.2 %; Fisher’s exact test, *p* = .003) or ever (7.4 % vs. 30.2 %; Fisher’s exact test, *p* = .001) being in recovery.

**Table 2:** Current drinking status, self-identified prior problem recognition and ‘in recovery’ status of former heavy drinkers in the UK or USA

	UK **(***n*** = 97)**	USA **(***n*** = 53)**	*p*
% Current moderate drinker (versus abstinent)	81.4	73.6	0.300
% Lifetime ICD10 alcohol dependence	32.9	35.0	0.841
% Had problem, now no longer do	24.1	56.0	0.001
% Major/very major problem when drinking at heaviest	10.3	32.1	0.002
% In recovery	4.2	21.2	0.003
% Ever in recovery	7.4	30.2	0.001

## Discussion

Participants from the USA were more likely than those from the UK to endorse that they used to have a problem with their drinking but now no longer do, and to regard themselves as currently or ever being in recovery. One possible explanation for this difference is that participants from the US may have had more severe alcohol use symptoms (or had them for longer, or more recently, or from a younger age) than those from the UK. This explanation cannot be ruled out using the current data. However, the proportion of participants who met criteria for alcohol dependence was similar in both the UK and the USA, reducing the likelihood that differences in the severity of alcohol consumption was the sole reason for the observed findings.

An alternate explanation for these findings is that these differences are not due to, or at least not entirely due to, differences between the two countries in the severity of alcohol consumption of participants prior to reducing to abstinence or moderate drinking. Mainly, recovery-oriented treatment services have been more common in the US than in the UK [7–9]. Given this preponderance of recovery treatment services in the US, former heavy drinkers in the US, compared to those from the UK, may be more likely to identify with being in recovery and, by extension, think about their drinking as having been a problem prior to reducing it. In addition, there are historical factors that may contribute to the extent to which the USA and UK accept concepts such as the value of abstinence as being a desirable goal to achieve [18]. Perhaps these historical cultural differences also influence the acceptance of an identity (having had a problem and now being in recovery) that is a central component of the AA approach to addressing alcohol concerns?

There are a number of limitations associated with the current data set that are important to address in order to strengthen the conclusions suggested by these analyses. Primarily, there is a need for a larger sample of former heavy drinkers in order to allow for the exploration of the association of demographic and drinking severity characteristics with identifying as being in recovery. Further, this larger sample should ideally be collected using methods that allow for confidence that the results are representative of the general population of former heavy drinkers in each country. In addition, there would be considerable worth in incorporating a qualitative component to such a study in order to establish what participants mean by being in recovery (or not) and whether this meaning varies by culture [19]. This qualitative component would also allow for a detailed exploration of the extent to which willingness (or unwillingness) to adopt a recovery identity might have implications for the most appropriate treatment models to promote in each country (i.e., sobriety or a harm reduction approach).

## Conclusions

A detailed understanding of the factors associated with a willingness to adopt a recovery identity could have considerable worth in informing ongoing policy decisions regarding the best ways to attract people with hazardous alcohol consumption to seek help and to then provide them with engaging tools to promote the successful resolution of their drinking concerns. While the current data provide preliminary support to suggest that former heavy drinkers in the US and UK may significantly differ in their identity as being in recovery, further work is necessary to generalize results at a population level. Additionally, future work should examine how different characteristics of previous heavy drinking (i.e. age of onset, duration) impact the recovery identities of former heavy drinkers in the USA and the UK.

## Data Availability

Available from the corresponding author on reasonable request.

## Abbreviation

AA: Alcoholics Anonymous
